# Virtual screening of phytochemicals from Indian medicinal plants against the endonuclease domain of SFTS virus L polymerase†

**DOI:** 10.1039/d1ra06702h

**Published:** 2022-02-22

**Authors:** R. P. Vivek-Ananth, Ajaya Kumar Sahoo, Ashutosh Srivastava, Areejit Samal

**Affiliations:** The Institute of Mathematical Sciences (IMSc) Chennai 600113 India asamal@imsc.res.in; Homi Bhabha National Institute (HBNI) Mumbai 400094 India; Discipline of Biological Engineering, Indian Institute of Technology Gandhinagar Gandhinagar 382355 India ashutosh.s@iitgn.ac.in

## Abstract

Severe fever with thrombocytopenia syndrome virus (SFTSV) causes a highly infectious disease with reported mortality in the range 2.8% to 47%. The replication and transcription of the SFTSV genome is performed by L polymerase, which has both an RNA dependent RNA polymerase domain and an N-terminal endonuclease (endoN) domain. Due to its crucial role in the cap-snatching mechanism required for initiation of viral RNA transcription, the endoN domain is an ideal antiviral drug target. In this virtual screening study for the identification of potential inhibitors of the endoN domain of SFTSV L polymerase, we have used molecular docking and molecular dynamics (MD) simulation to explore the natural product space of 14 011 phytochemicals from Indian medicinal plants. After generating a heterogeneous ensemble of endoN domain structures reflecting conformational diversity of the corresponding active site using MD simulations, ensemble docking of the phytochemicals was performed against the endoN domain structures. Apart from the ligand binding energy from docking, our virtual screening workflow imposes additional filters such as drug-likeness, non-covalent interactions with key active site residues, toxicity and chemical similarity with other hits, to identify top 5 potential phytochemical inhibitors of endoN domain of SFTSV L polymerase. Further, the stability of the protein–ligand docked complexes for the top 5 potential inhibitors was analyzed using MD simulations. The potential phytochemical inhibitors, predicted in this study using contemporary computational methods, are expected to serve as lead molecules in future experimental studies towards development of antiviral drugs against SFTSV.

## Abstract 2

Virtual screening of a large phytochemical library from Indian medicinal plants to identify potential endonuclease inhibitors against emerging virus SFTSV.

## Graphical abstract

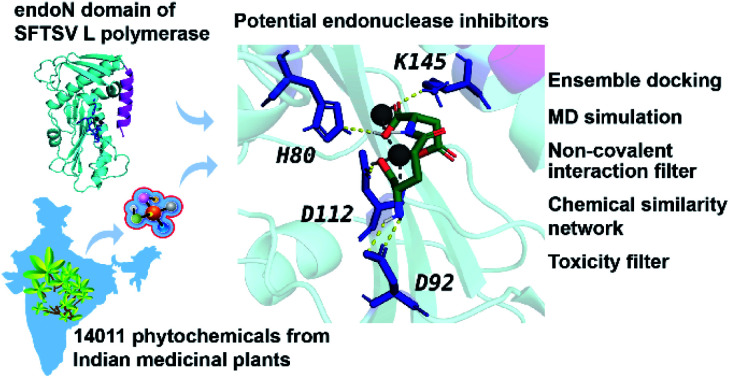

## Introduction

SFTSV, also known as *Huaiyangshan banyangvirus*, is a segmented negative-sense RNA virus (sNSV)^1,2^ that causes severe fever with thrombocytopenia syndrome (SFTS). Although SFTS is suspected to be a tick-borne disease,^3^ human-to-human transmissions through direct contact with contaminated blood or tissue samples of infected person have also been reported.^4–6^ SFTS is characterized by clinical symptoms such as high fever, thrombocytopenia, leukopenia, gastrointestinal disorder and multiple organ dysfunction.^2,7^ The first cases of SFTS were reported from China in 2009,^8^ and thereafter, cases have also been reported from Japan, Korea, Vietnam and Taiwan.^3^ The reported mortality from SFTSV infection varies considerably across countries. While Japan and South Korea have reported a high mortality rate of 27% and 23.3%, respectively, China has reported a comparatively lower mortality rate of 6.18%.^3^ Notably, the World Health Organization (WHO) has included SFTS in its 2018 blueprint on diseases which pose major public health risk due to their epidemic potential, and therefore, immediate scientific attention is warranted towards development of novel therapies and vaccines to combat SFTSV infection.^9,10^

Apart from SFTSV, other sNSVs include influenza virus, Rift Valley fever virus, Hantaan virus and Crimean–Congo hemorrhagic fever virus.^2,11^ Notably, the segmented genome of these viruses provide an evolutionary advantage towards emergence of new pathogenic strains through reassortment of genomic segments.^11,12^ SFTSV genome consists of 3 negative-sense RNA segments namely, L (large), M (medium) and S (small) with 6368 nucleotides (nt), 3378 nt and 1746 nt, respectively.^8,13,14^ The L segment encodes a multifunctional and multidomain, 2084 amino acids (aa) long L polymerase.^2^ The M segment encodes for 516 aa long glycoprotein (Gn) and 511 aa long glycoprotein (Gc). The S segment encodes for 293 aa long non-structural protein (Ns) and 245 aa long nucleoprotein (N).^14^ SFTSV L polymerase consists of 3 functional domains and 2 structural domains.^15^ The 3 functional domains consist of an endonuclease (endoN) domain present in the N-terminus, an RNA-dependent RNA polymerase (RdRp) domain, and a cap-binding domain (CBD) in the C-terminus.^15^ The 2 structural domains consist of an arm domain with a blocker motif and a C-terminal lariat domain.^15^ The sNSVs including SFTSV, require L polymerase for genome replication and viral transcription, but the sNSVs do not encode a domain for the synthesis of 5′-cap structure for their mRNA to initiate viral transcription.^2,16,17^ Instead, viral transcription is initiated by a cap-snatching mechanism with active involvement of the endoN and the CBD. The CBD acts as a recognition apparatus and binds to the 5′ capped structure of the host mRNA. The endoN cleaves the capped structure and attaches it to the viral mRNA. Thereafter, the transcription is further processed by the host ribosome.^2,16–18^ The active site of the endoN contains divalent Manganese (Mn^2+^) ions which are crucial for its activity.^2,16–18^ The endoN has structure- and sequence-wise similarity with endonucleases of other bunyaviruses and influenza virus.^2,17^ In terms of both structure and function, the endoN is the most characterized domain of L polymerase, and is functional by itself.^2,18^ The above factors make the endoN an important antiviral drug target against SFTSV.^2,16,17,19^

Previously, Wang *et al.*^2^ have shown using enzymatic assays that Baloxavir acid (BXA), which is an active form of the US Food and Drug Administration (FDA) approved prodrug Baloxavir marboxil, and the known endonuclease inhibitor L-742001, inhibited the activity of the endoN.^2^ To further expedite efforts toward identification and development of antiviral drugs against SFTSV, computer-aided drug design (CADD) methods such as molecular docking and molecular dynamics (MD) simulations can be used in tandem with *in vitro* and *in vivo* experiments.^20^

According to a WHO report, majority of the Indian rural population use Ayurveda and medicinal plants for their primary healthcare.^21^ Since the discovery of morphine, a plant-based compound first used as a drug in the year 1826, scientists have tried to isolate, modify and screen many plant-derived compounds or phytochemicals for the treatment of different diseases.^22,23^ For instance, drugs made from artemisinin, a phytochemical derived from the medicinal plant *Artemisia annua*, have been used for the treatment of multidrug-resistant malaria.^24^ In this context, some of the authors of this study have previously built IMPPAT, the largest knowledgebase on phytochemicals from Indian medicinal plants.^25^ The compiled information in IMPPAT,^25^ in particular the largest collection of curated natural products from Indian medicinal plants, can be used to accelerate natural product based drug discovery.^26–32^ In this direction, we have performed a virtual screening of 14 011 phytochemicals from Indian medicinal plants against the endoN, using ensemble molecular docking. Since the divalent Mn^2+^ ions act as co-factors for the enzymatic activity of the endoN domain,^2^ both molecular docking and MD simulations were performed with two divalent Mn^2+^ ions present in the active site of the endoN. Lastly, we have also demonstrated the stability of the protein–ligand docked complexes for the top 5 potential phytochemical inhibitors identified in this study using MD simulations.

## Methods

### Screening library of phytochemicals from Indian medicinal plants

In this study, we have used 14 011 phytochemicals from Indian medicinal plants compiled from the IMPPAT database^25^ and other literature sources.^33–48^ Notably, IMPPAT is the largest phytochemical database for herbs used in traditional Indian medicine, and has been serving as a resource for antiviral drug discovery.^31,49–52^ The compiled library of 14 011 phytochemicals were filtered for drug-likeness using Lipinski's rule of five (RO5).^53^ Based on RO5, 10 510 phytochemicals were found to be drug-like. The three-dimensional (3D) structures of these 10 510 drug-like phytochemicals were retrieved from PubChem.^54,55^ Thereafter, the retrieved 3D chemical structures were energy-minimized with OpenBabel's^56^ obminimize command using MMFF94 force field. Finally the energy-minimized 3D chemical structures were converted from SDF file format to PDB file format using OpenBabel.^56^ Subsequently, the energy-minimized structures for the 10 510 drug-like phytochemicals were used for virtual screening.

### Preparation of the endoN domain structure with Mn^2+^ ions in the active site

**Fig. 1:**
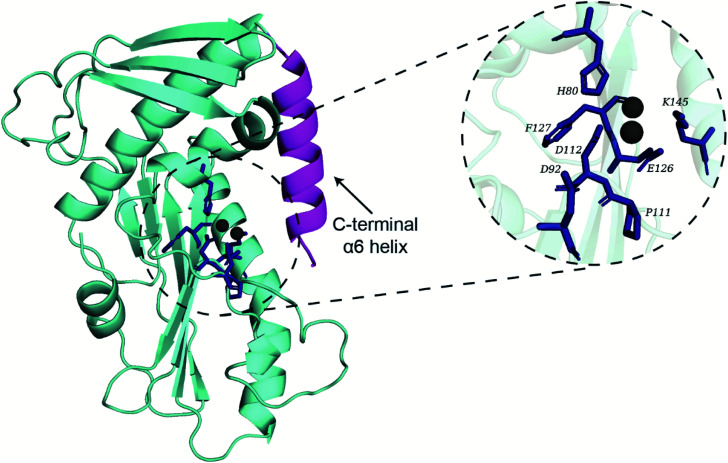
Cartoon representation of the prepared crystal structure of the endoN domain of SFTSV L polymerase. The active site residues important for the endonuclease activity are shown as sticks colored in deep blue along with the pair of divalent Mn^2+^ ions shown as spheres colored in grey. The active site amino acid residues, H80, D92, P111, D112, E126, E127 and K145, important for activity of the endoN domain are shown in the expanded view. The C-terminal α6 helix of the endoN domain is shown in magenta color.

Recently, Wang *et al.*^2^ have published the X-ray crystal structure of the endoN (PDB 6NTV) with resolution of 2.40 Å which we obtained from the Protein Data Bank (https://www.rcsb.org/). However, the divalent Mn^2+^ ions required for the activity of endoN were absent in the published structure (PDB 6NTV). Therefore, two Mn^2+^ ions were placed in the endoN structure (PDB 6NTV) by aligning it to the structure of the influenza A virus H1N1 polymerase subunit endonuclease in complex with 2,4-dioxo-4-phenylbutanoic acid (PDB 4AWF). This alignment was performed in PyMOL^57^ using residues 112–130 of PDB 6NTV and residues 108–123 of PDB 4AWF. Lastly, the selenomethionine residues (MSE) in PDB 6NTV were replaced with methionine residues (MET) using UCSF Chimera Dock Prep tool.^58^ This prepared endoN structure with Mn^2+^ ions in the active site (Fig. 1) was used for docking and MD simulations.

### MD simulations of the endoN domain with Mn^2+^ ions in the active site

Using GROMACS 5.1.5 ^59^ with Amber03 force field,^60^ we performed MD simulation of the prepared endoN structure. The force field parameters for the Mn^2+^ ions were retrieved from http://amber.manchester.uk/. The prepared structure was placed at the center of a dodecahedron box with periodic boundary condition and minimum distance of 12 Å from the box edge. TIP3P water model was used to solvate the system, and thereafter, the system was neutralized by addition of 12 sodium (Na^+^) ions. To ensure that the Mn^2+^ ions remain in the active site of the endoN, distance restraint of 1000 kJ mol^−1^ nm^−2^ was imposed between the Mn^2+^ ions and the oxygen atoms of the residues D112 and E126, and the nitrogen atom of the residue H80 in the active site.

The above-mentioned system was energy minimized using steepest descent algorithm, with energy minimization tolerance set at 100 kJ mol^−1^ nm^−1^. The system was then subjected to 1 ns *NVT* equilibration simulation with 2 fs time step and temperature set at 300 K. Next, the system was subjected to 1 ns *NPT* simulation with 2 fs time step to equilibrate the pressure of the system to 1 bar. Note that the positions of the heavy atoms of the protein and Mn^2+^ ions were restrained using a force constant of 1000 kJ mol^−1^ nm^−2^ during the *NVT* and the *NPT* simulation. The bond lengths were constrained using LINCS algorithm.^61^ Thereafter, a final equilibration simulation was performed for 1 ns with 2 fs time step after removing the position restraint and placing distance restraint on the Mn^2+^ ions.

Finally, the equilibrated system was simulated for 270 ns in quintuplicate (5 replicas). The v-rescale temperature^62^ and Parrinello–Rahman pressure coupling method^63^ were used for maintaining the system temperature at 300 K and pressure at 1 bar during the 270 ns simulation.

### Clustering and extraction of representative structures for ensemble docking

The protein trajectories from 50 ns to 270 ns from each of the quintuplicate simulations were combined and 10000 frames were extracted at equal interval. The extracted frames were superimposed on the first frame of the combined trajectory, using Cα atoms of the residues which are part of the protein secondary structure, except for the α6 helix as reference. The α6 helix was earlier shown to be considerably dynamic,^2^ an observation which was also confirmed in the MD simulations performed here (see Results). Consequently, the α6 helix was excluded from clustering calculations. Clustering of the 10000 frames was performed using Jarvis–Patrick method^59,64^ by considering only the heavy atoms of the active site residues. This resulted in the identification of 322 clusters with top 25 clusters accounting for more than 66% of the total frames (*i.e.*, 6648 frames). Thereafter, 25 protein structures corresponding to the centers of the top 25 clusters were retrieved. For subsequent ensemble docking, we have used the prepared endoN crystal structure and the above-mentioned 25 structures obtained from the top 25 clusters.

### MD simulations of the endoN domain in complex with phytochemical hits

After ensemble docking, the protein–ligand docked complexes for the top 5 inhibitors were subjected to MD simulations of 100 ns to assess their stability. The Generalized Amber Force Field (GAFF) parameters of the ligands were determined using the antechamber^65^ implemented *via* acpype program.^66^ The partial charges on ligand atoms were determined by first calculating the electrostatic potential (ESP) for the ligand molecule in Gaussian v9 ^67^ using Density Functional Theory BLYP/6-31G* level. This was followed by RESP charge calculation using antechamber. The MD simulation of the protein–ligand docked complex was performed following the same procedure as described above for the prepared endoN structure with Mn^2+^ ions in the active site.

From the MD simulation trajectories, the radius of gyration (*R*_g_), root mean square deviation (RMSD) and root mean square fluctuation (RMSF) of the protein or ligand atoms were computed using GROMACS.

### Molecular docking of phytochemicals against the endoN structure

We have used AutoDock 4.2.6 software optimized for GPU, also known as AutoDock-GPU,^68^ to perform docking of the 10510 drug-like phytochemicals against the prepared endoN structure and 25 structures obtained from clustering of the combined MD simulation trajectory.

For ligand docking, we used AutoGrid 4.2.6 ^69^ to create a grid of 60 × 60 × 65 grid points with a spacing of 0.375 Å, centered based on the backbone atoms of the key active site residues namely, H80, D112, E126, F127, S128, K145, E219 and A223, of the endoN.^2^ Here, the scripts prepare_receptor4.py, prepare_ligand4.py and prepare_gpf4.py in AutoDockTools^69^ were used for preparing protein structure, preparing ligand structure and creating grid parameter file, respectively. Further, a +2 charge was assigned to the two Mn atoms in the prepared protein structure file in PDBQT format.

Subsequently, docking was performed with default parameters in AutoDock-GPU which makes use of Lamarckian genetic algorithm and gradient-based local search method ADELTA.^68^ Each AutoDock4-GPU docking run gives 20 docked conformations of the ligand with their respective binding energies with the specified protein structure. From the output of AutoDock4-GPU, the best docked conformation of the ligand (with lowest binding energy) was selected, and the corresponding protein–ligand complex was generated using custom python scripts and pdb-tools.^70^ Afterwards, we used custom scripts described in our previous publication^31^ to analyze the protein–ligand docked complexes, and identify the ligand binding site residues and the non-covalent interactions between protein residues and ligand.

### Chemical similarity network of top phytochemical inhibitors

A chemical similarity network (CSN) of the top inhibitors of endoN domain was constructed wherein nodes correspond to phytochemicals and edges indicate high chemical similarity between pairs of phytochemicals. The extent of similarity between pairs of phytochemicals was quantified using Tanimoto coefficient (*T*_c_) with ECFP4 fingerprint.^71,72^*T*_c_ values for any pair of chemicals can range from 0 (implying no or very low similarity) to 1 (implying exact or very high similarity). Here, we have used a *T*_c_ threshold of ≥0.5 to specify the edges in the CSN. The CSN was visualized using Cytoscape.^73^

### Physicochemical, drug-likeness and predicted ADMET properties of top inhibitors

For the top phytochemical inhibitors, we have computed the physicochemical, drug-likeness and predicted ADMET (absorption, distribution, metabolism, excretion and toxicity) properties using RDKit,^72^ SwissADME^74^ and ProTox-II.^75^ Further, the chemical classification of the top inhibitors was predicted using ClassyFire^76^ (http://classyfire.wishartlab.com/).

### MM-GBSA calculation

Molecular mechanics with generalized Born and surface area solvation (MM-GBSA) method was used to compute the binding energy of the top 5 inhibitors (L1–L5) identified here. For each of these 5 inhibitors, 51 frames were extracted at 1 ns interval from the 50 ns to 100 ns region of the 100 ns MD simulation trajectory of the corresponding protein–ligand complex, namely endoN–L1, endoN–L2, endoN–L3, endoN–L4 and endoN–L5. Thereafter, we employed gmx_MMPBSA^77^ which uses MMPBSA.py^78^ of AMBER, to compute MM-GBSA based binding energies from the extracted frames and GROMACS trajectories. For this, the generalized Born method (igb) was set to 8, the PBRadii was set to 4 and the internal dielectric constant was set to 10.

## Results and discussion

### Conformational dynamics of the endonuclease domain of SFTSV L polymerase

**Fig. 2:**
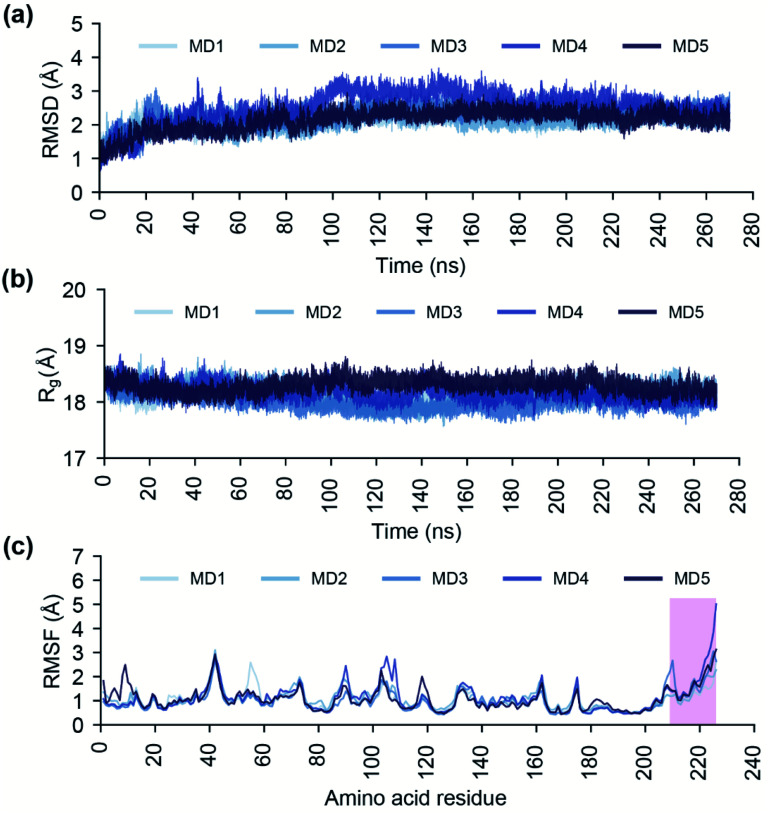
Analysis of the endoN trajectories from the 270 ns MD simulations in quintuplicate. (a) RMSD of the Cα atoms of all protein residues and Mn^2+^ ions. (b) Radius of gyration (*R*_g_) of the complete protein structure. (c) RMSF of the Cα atoms of all protein residues. In this plot, a vertical box spanning the protein residues 209–226 highlights the RMSF values of the protein residues of C-terminal α6 helix of the endoN domain.

The endoN has a canonical structure similar to other cap-snatching endonucleases, with six α helices surrounding a six strand β sheet (Fig. 1).^2^ The key active site residues, H80, D92, P111, D112, E126, F127 and K145, of the endoN are conserved, and the residues H80, D112 and E126 are structurally poised to coordinate Mn^2+^ for catalytic activity (Fig. 1). To study the conformational dynamics of the endoN and generate heterogeneous ensemble for molecular docking, we performed MD simulation of the corresponding prepared structure for 270 ns in quintuplicate (Methods). The RMSD of the Cα atoms of the endoN residues and Mn^2+^ ions remain stable during the MD simulations with RMSD values largely within 3 Å (Fig. 2a). Specifically, the RMSD values from the quintuplicate simulations (MD1–MD5 in Fig. 2a) of the endoN show fewer fluctuations after 230 ns. Further, the radius of gyration (*R*_g_) shows little variation (MD1–MD5 in Fig. 2b). This indicates that the endoN domain structure remains compact during the MD simulations (Fig. 2b). Lastly, the RMSF of the amino acid residues in the endoN reveals the protein regions that exhibit substantial dynamics, and the corresponding plot confirms the expected substantial dynamics in the C-terminal α6 helix region during MD simulations (Fig. 2c). In sum, we observe that all regions of the endoN, with the exception of the C-terminal α6 helix, remain stable during the MD simulations, and these observations are in concordance with previous observations made by Wang *et al.*^2^

### Identification of potential phytochemical inhibitors of the endoN domain

**Fig. 3:**
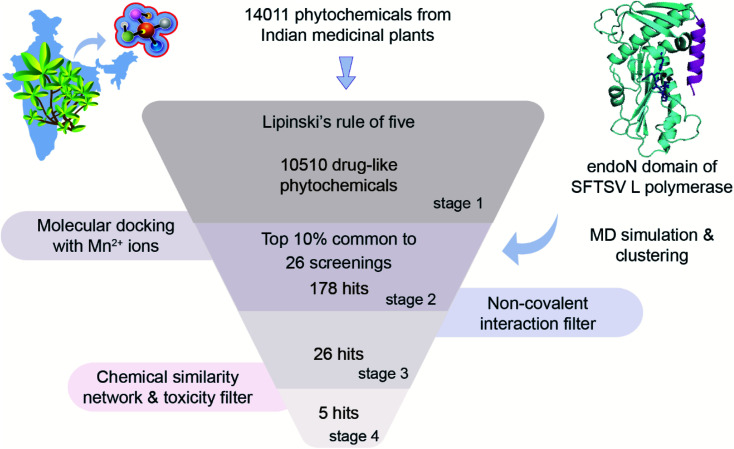
Four-stage virtual screening workflow for the identification of potential phytochemical inhibitors of the endoN domain of SFTSV L polymerase.

In this study, we have implemented a four-stage virtual screening workflow to identify potential phytochemical inhibitors of the endoN domain of SFTSV L polymerase (Fig. 3).

Firstly, the natural product library of 14 011 phytochemicals from Indian medicinal plants was filtered for drug-likeness using the Lipinski's RO5 ^53^ (Methods), and this led to a subset of 10 510 drug-like phytochemicals at the end of stage 1.

Secondly, we used AutoDock-GPU to perform docking of the 10 510 drug-like phytochemicals against 26 structures of the endoN (Methods). Since the Mn^2+^ ions are crucial for the endonuclease activity of SFTSV L polymerase,^2,18^ we performed docking with the two Mn^2+^ ions in the active site. For each of the 26 structures of the endoN, we determined the top 10% of phytochemical hits based on the binding energies obtained *via* docking. Thereafter, we determined the set of phytochemicals which are common to the top 10% hits for each of the 26 structures, and this led to a subset of 178 phytochemicals at the end of stage 2.

Thirdly, for these 178 phytochemicals, we determined the non-covalent interactions between the ligand and the binding site residues in their best docked pose with each of the 26 structures (Methods). Thereafter, we selected phytochemicals which can: (a) bind to or interact with at least 4 out of the 7 key active site residues (H80, D92, P111, D112, E126, F127 and K145) in the best docked pose with the prepared crystal structure of the endoN, and (b) bind to or interact with at least 4 out of the 7 key active site residues in the best docked poses for at least 90% of the 25 structures of the endoN obtained from clustering of the MD simulation trajectory. This filter based on ligand interactions with key active site residues led to identification of 26 phytochemicals as potential inhibitors of the endoN at the end of stage 3 (ESI Table S1†).

**Fig. 4:**
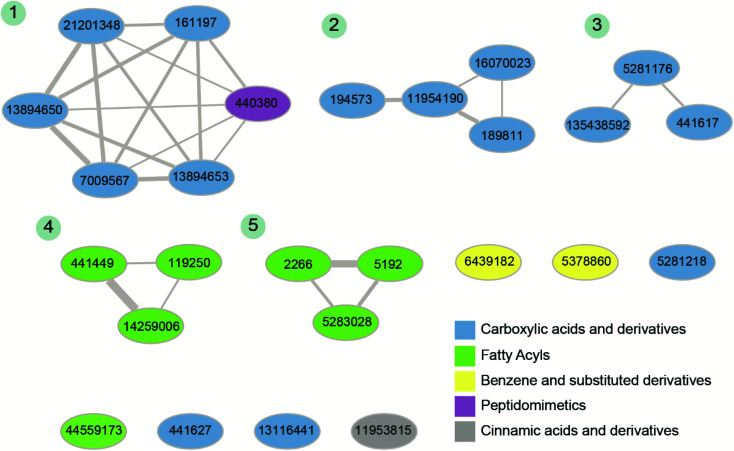
Chemical similarity network (CSN) of the 26 phytochemical inhibitors identified at the end of stage 3 of the virtual screening workflow (Fig. 3). The nodes correspond to phytochemicals which are labeled by their PubChem identifiers and are colored based on their chemical class predicted by ClassyFire. The edge thickness shows the extent of chemical structure similarity between the phytochemicals. The connected components of the CSN are labeled from 1 to 5 based on their size.

**Fig. 5:**
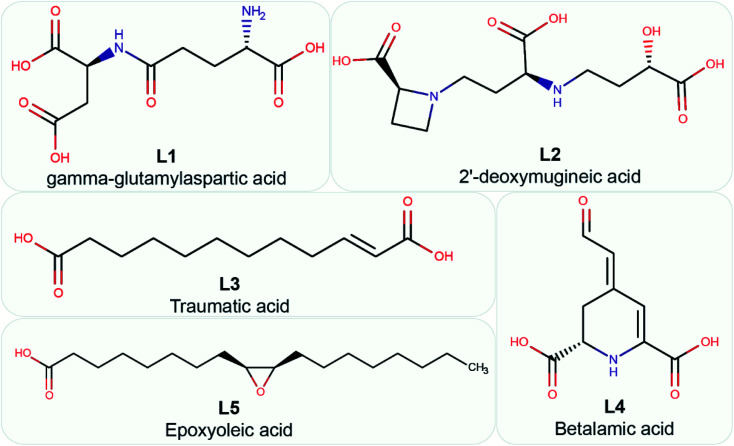
Chemical name and 2D structure for the top 5 phytochemical inhibitors of the endoN identified in this study.

Fourthly, for the 26 phytochemical inhibitors at the end of stage 3, we constructed a chemical similarity network (CSN) to shortlist hits with unique chemical structure (Fig. 4; Methods). The phytochemicals in the CSN are labeled by their PubChem identifiers and are colored based on their chemical class predicted by ClassyFire.^76^ Among these 26 phytochemicals, 15 were predicted to be ‘carboxylic acids and derivatives’, 7 were predicted to be ‘fatty acyls’, 2 were predicted to be ‘Benzene and substituted derivatives’, 1 was predicted to be ‘peptidomimetics’ and 1 was predicted to be ‘cinnamic acids and derivatives’ (Fig. 4 and ESI Table S1†). Notably, the CSN partitions the 26 phytochemicals into 5 connected components (clusters) and 7 isolated nodes based on similarity or dissimilarity of their structures (Fig. 4). Further, each connected component in the CSN typically consists of phytochemicals belonging to a single chemical class, with the exception being the largest connected component of size 6, which has 5 phytochemicals belonging to class ‘carboxylic acids and derivatives’ and 1 phytochemical belonging to class ‘peptidomimetics’ (Fig. 4). Subsequently, we considered only the chemical classes that have more than one phytochemical among the 26 phytochemicals, and this led to a subset of 24 phytochemicals belonging to chemical classes ‘carboxylic acids and derivatives’, ‘fatty acyls’ and ‘benzene and substituted derivatives’ (ESI Table S2†). The toxicity of these 24 phytochemicals was assessed using ProTox-II,^75^ and this led to a subset of 12 phytochemicals which were predicted to be non-toxic (class: 5 or class: 6) (ESI Table S2†). Interestingly, the 12 phytochemicals predicted to be non-toxic, were found to be distributed across 5 connected components of the CSN, and a final subset of 5 phytochemical inhibitors, one each from the 5 connected components based on the lowest docking binding energy, were chosen for further analysis. The top 5 potential phytochemical inhibitors of the endoN domain are: gamma-glutamylaspartic acid (L1), 2′-deoxymugineic acid (L2), traumatic acid (L3), betalamic acid (L4) and epoxyoleic acid (L5) (Fig. 5; Table 1 and ESI Table S3†). Notably, we find that all of the top 5 inhibitors (L1–L5) have much lower docking binding energy with the endoN in comparison to the docking binding energy value of −7.3 kcal mol^−1^ obtained for the known inhibitor Baloxavir (BXA).

**Table 1:** Binding energy and plant source for the top 5 phytochemical inhibitors (L1–L5) of the endoN identified in this study. For each inhibitor, the table gives the phytochemical symbol, PubChem identifier, chemical name, docking based binding energy and MM-GBSA based binding energy in kcal mol^−1^ with the prepared crystal structure, and plant source

Phytochemical symbol	PubChem identifier	Chemical name	Docking binding energy (kcal mol^−1^)	MM-GBSA binding energy (kcal mol^−1^)	Plant source
L1	CID: 161197	Gamma-glutamylaspartic acid	−22.01	−27.23 ± 5.57	*Vigna mungo*
L2	CID: 189811	2′-Deoxymugineic acid	−20.99	−44.83 ± 3.33	*Oryza sativa*
L3	CID: 5283028	Traumatic acid	−18.77	−38.00 ± 5.14	*Phaseolus vulgaris*
L4	CID: 5281176	Betalamic acid	−15.35	−16.61 ± 3.96	*Opuntia ficus-indica*
L5	CID: 119250	Epoxyoleic acid	−15.16	−40.66 ± 7.67	*Abelmoschus ficulneus*; *Hibiscus sabdariffa*; *Hibiscus caesius*; *Petroselinum crispum*; *Shorea robusta*

### Description of the top 5 phytochemical inhibitors of the endoN domain

Fig. 5 displays the 2D chemical structures of the top 5 phytochemical inhibitors (L1–L5) of the endoN identified here. Based on the chemical class predicted by ClassyFire,^76^ phytochemicals L1, L2 and L3 are ‘carboxylic acids and derivatives’, whereas phytochemicals L4 and L5 are ‘fatty acyls’.

**Fig. 6:**
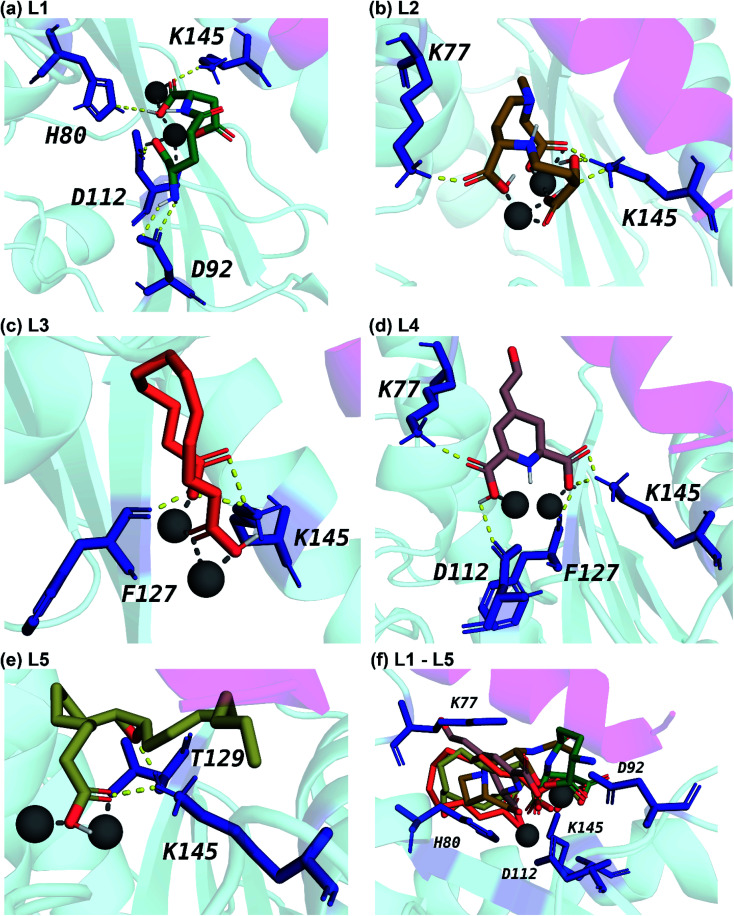
Cartoon representation of the interactions between the ligand and the protein residues in the best docked pose of the top 5 phytochemical inhibitors (L1–L5) with the endoN. The carbon atoms of (a) L1 are in seagreen, (b) L2 are in darkgoldenrod, (c) L3 are in tomato, (d) L4 are in rosybrown, and (e) L5 are in darkkhaki color. (f) This subfigure shows the docked pose of L1–L5 in the active site of endoN in a single frame. The oxygen and nitrogen atoms of the inhibitors are in red and blue color, respectively. The protein residues involved in hydrogen bond interactions with the inhibitors are shown as sticks colored in deepblue. The Mn^2+^ ions are shown as grey colored spheres. The hydrogen bond interactions are shown as yellow colored dashed lines and the interaction with the Mn^2+^ ions are shown as grey colored dashed lines.

**Fig. 7:**
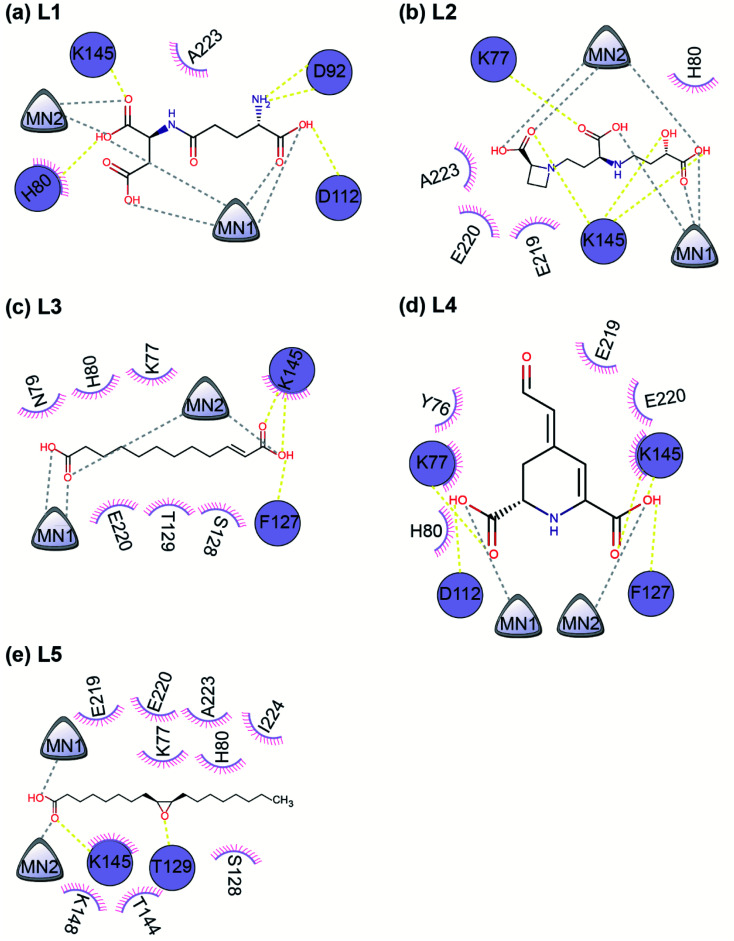
A 2D representation of the interactions between the ligand and the protein residues in the best docked pose of the top 5 phytochemical inhibitors (L1–L5) with the endoN. The protein residues involved in hydrogen bond interactions with the inhibitors are shown as circles colored in deepblue. The protein residues involved in hydrophobic interactions with the inhibitors are shown as short circle segments with spikes. The Mn^2+^ ions are shown as triangles. The hydrogen bond interactions are shown as yellow colored dashed lines and the interaction with the Mn^2+^ ions are shown as grey colored dashed lines. In this schematic figure, the protein residues involved in hydrogen bond interactions or hydrophobic interactions with the inhibitors have been placed manually around the 2D chemical structure of the ligand.

All five inhibitors engage in extensive electrostatic interactions with the divalent Mn^2+^ ions in the active site of the endoN domain (Fig. 6 and 7). Considering the importance of Mn^2+^ in substrate binding, the inhibitors identified here reveal themselves as potential competitive inhibitors. The carboxylic group present in all five inhibitors interacts with the active site residue K145 *via* hydrogen bonds (Table 2; Fig. 6 and 7). The ligands also engage in non-polar interactions with the active site residue H80 (Table 2 and Fig. 7).

**Table 2:** Non-covalent interactions for top 5 phytochemical inhibitors (L1–L5) with the prepared crystal structure of the endoN in the best docked pose. For each protein–ligand complex, the table lists the number of hydrogen bonds, the residues in the ligand binding site, and the residues forming hydrogen bond and hydrophobic interactions with the ligand atoms. Note that the hydrophobic interactions listed here are between the carbon atom of the protein residue and the carbon, halogen or sulfur atom of the ligand

Protein–ligand complex	Number of hydrogen bonds	Binding site residues	Hydrogen bond interaction residues	Hydrophobic interaction residues
endoN–L1	5	H80, D92, T110, D112, E126, F127, K145, K148, E219, A223	H80, D92, D112, K145	H80, A223
endoN–L2	4	K77, H80, T110, D112, E126, F127, K145, K148, E219, E220, A223	K77, K145	H80, E219, E220, A223
endoN–L3	3	K77, H80, D112, F127, S128, T129, K145, E219, E220	F127, K145	K77, N79, H80, S128, T129, K145, E220
endoN–L4	5	Y76, K77, H80, D112, F127, K145, K216, E219, E220	K77, D112, F127, K145	Y76, K77, H80, K145, E219, E220
endoN–L5	2	K77, H80, D112, E126, F127, S128, T129, T144, K145, K148, E219, E220, A223, I224	T129, K145	K77, H80, S128, T144, K145, K148, E219, E220, A223, I224

Phytochemical L1 (gamma-glutamylaspartic acid), a dipeptide made of gamma-glutamate and aspartic acid, is produced by the herb *Vigna mungo* (black gram). In endoN–L1 complex, the three carboxyl groups surround Mn-1 ion forming electrostatic interactions (Fig. 6 and 7). L1 binding is further stabilized by hydrogen bond (H-bond) interactions with the active site residues H80, D92, D112 and K145 (Fig. 6).

Phytochemical L2 (2′-deoxymugineic acid) is a phytosiderophore that can act as an iron-chelator. L2 is produced by *Oryza sativa* (rice) and is found in the phloem sap in complex with iron.^79^ Similar to L1, the carboxyl groups in L2 also interact with Mn-1 in endoN–L2 complex, and L2 binding is further stabilized by H-bond interactions with surrounding K77 and K145 (Fig. 6 and 7). Additionally, L2 also makes several non-polar interactions with the binding site residues (Table 2 and Fig. 7).

Phytochemical L3 (traumatic acid) is a monosaturated dicarboxylic acid that has a role in wound healing in plants. It was first isolated from *Phaseolus vulgaris* (common bean). It has been shown to reduce proliferation in breast cancer cells and reduce the production of reactive oxygen species (ROS) in fibroblasts.^80,81^ In endoN–L3 complex, L3 forms a hairpin-like structure within the binding pocket enabling both carboxyl groups to form electrostatic interactions with the two Mn^2+^ ions (Fig. 6). The carboxyl oxygens also form H-bonds with main chain of F127 and side chain of K145 (Fig. 6 and Table 2). The 10-carbon aliphatic chain engages in multiple hydrophobic interactions with the binding pocket residues such as H80, T129 and N79 (Table 2 and Fig. 7).

Phytochemical L4 (betalamic acid) is the core structural unit of a class of plant pigments called betalain found in plants of the order *Caryophyllales*.^82^ Betalain are known to have antioxidant and free radical scavenging properties.^83^ In endoN–L4 complex, the two carboxyl groups in L4 interact with an Mn^2+^ ion each and also form H-bonds with K77, K145 and D112 and F127 (Fig. 6 and Table 2). The compound also engages in van der Waals interaction with binding pocket residues such as Y76 and H80 (Fig. 7 and Table 2).

Phytochemical L5 (epoxyoleic acid) is an epoxy fatty acid produced by herbs *Abelmoschus ficulneus*, *Hibiscus sabdariffa*, *Hibiscus caesius*, *Petroselinum crispum* and *Shorea robusta*. In endoN–L5 complex, L5 takes up a bent conformation similar to L3 with the carboxyl group making electrostatic interaction with the Mn^2+^ ions and H-bonds with K145 (Fig. 6). The epoxy oxygen makes a H-bond with the main chain of T129 (Fig. 6 and Table 2). The long aliphatic chain makes several van der Waals interactions with binding pocket residues (Fig. 7 and Table 2).

In comparison, the known inhibitor BXA docked with the endoN, did not interact with the Mn^2+^ ions in the active site (ESI Fig. S1†). BXA is stabilized by extensive H-bond interactions and other non-covalent interactions with the active site residues of the endoN, including H-bond with side chain of K145 (ESI Fig. S1†). The two fluorine atoms of BXA form halogen bonds, one each with K145 and A223 (ESI Fig. S1†). The sulfur atom of BXA forms a chalcogen bond with S128 (ESI Fig. S1†). Additionally, BXA also makes hydrophobic interactions with the binding site residues (ESI Fig. S1b†). Note that the docked binding pose of BXA with endoN domain structure (ESI Fig. S1a†) differs from the docked binding pose reported by Wang *et al.*^2^ It is worth mentioning that the residues H80, D92, D112, E126, F127 and K145 are considered either important for metal binding or endonuclease activity of the endoN domain of SFTSV L polymerase.^2^ The analysis of protein–ligand complexes of the top 5 phytochemical inhibitors reveal key interactions that could be responsible for high docking score across different conformations of endoN.

### MD analysis of the protein–ligand complexes of the top 5 phytochemical inhibitors

**Fig. 8:**
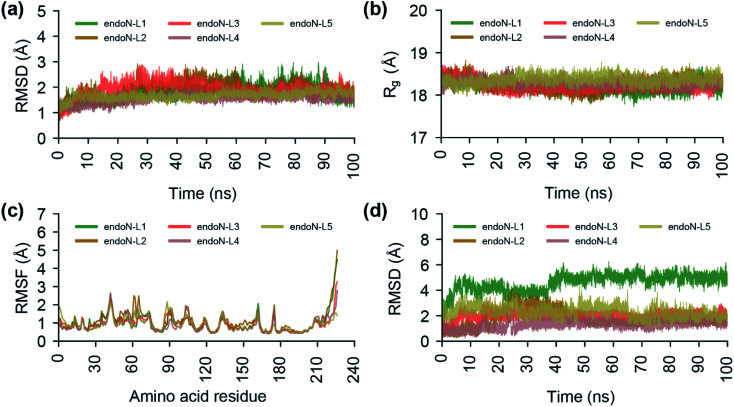
Analysis of the trajectories from the 100 ns MD simulations of the protein–ligand docked complexes of the top 5 phytochemical inhibitors (L1–L5) with the endoN. (a) RMSD of the Cα atoms of all the protein residues and Mn^2+^ ions. (b) Radius of gyration (*R*_g_) of the complete protein structure. (c) RMSF of the Cα atoms of all protein residues. (d) RMSD of the heavy atoms of the ligands L1–L5.

To study the stability of the docked complexes, we have performed a 100 ns MD simulation of the protein–ligand docked complexes of the top 5 phytochemical inhibitors (L1–L5) with the prepared crystal structure of endoN (Fig. 8; Methods). All five protein–ligand complexes remain stable during 100 ns MD simulations with the average RMSD values of the Cα atoms and Mn^2+^ ions for endoN–L1 = 1.82 ± 0.32 Å, endoN–L2 = 1.89 ± 0.34 Å, endoN–L3 = 1.89 ± 0.30 Å, endoN–L4 = 1.53 ± 0.17 Å and endoN–L5 = 1.66 ± 0.15 Å (Fig. 8a). The average *R*_g_ also shows only small deviation throughout the trajectory with the values for endoN–L1 = 18.19 ± 0.12 Å, endoN–L2 = 18.28 ± 0.15 Å, endoN–L3 = 18.22 ± 0.12 Å, endoN–L4 = 18.29 ± 0.09 Å and endoN–L5 = 18.37 ± 0.10 Å (Fig. 8b). This indicates that ligand binding does not change the structure and compactness of the endoN considerably. The flexibility of the residues in protein–ligand complexes, as depicted by the RMSF values, also closely follow the RMSF values for residues in the MD simulation of the apo protein (Fig. 2c and 8c). The ligands, however, show considerable variability in terms of their stability within the binding pocket (Fig. 8d). The RMSD value of the heavy atoms of L1 stabilizes after 40 ns, with the average RMSD of 5.02 ± 0.30 Å for frames between 40 and 100 ns.

The visual inspection of the MD simulation trajectory of endoN–L1 reveals that the carboxyl groups of aspartic acid in L1 continue to interact with the Mn-1 ion till 40 ns, post which it moves away from Mn-1 and stabilizes. On the other hand, the two carboxyl groups of gamma-glutamate in L1 continue their interaction with Mn-1 and Mn-2 till 100 ns. For the other four ligands, the heavy atom RMSD fluctuates around 2 Å (endoN–L2 = 1.72 ± 0.67 Å, endoN–L3 = 1.99 ± 0.30 Å, endoN–L4 = 1.31 ± 0.32 Å and endoN–L5 = 2.30 ± 0.42 Å). The key interactions between ligands and the binding site residues such as those between the carboxyl group and Mn^2+^ ions and hydrogen bond with K145 remain stable during the MD simulations.

We also performed a 100 ns MD simulation of the protein–ligand docked complex of BXA with the endoN domain of SFTSV L polymerase. The average RMSD of the Cα atoms and Mn^2+^ ions (1.85 ± 0.33 Å) and the average *R*_g_ of the protein (18.27 ± 0.13 Å) for endoN–BXA complex are similar to average RMSD and *R*_g_ values from MD simulation of the docked complexes of the top 5 inhibitors (ESI Fig. S2a, b†; 8a and b). The RMSF values of the protein residues for endoN–BXA complex also closely follows the RMSF values from MD simulation of the docked complexes of the top 5 inhibitors (ESI Fig. S2c;† and 8c). The RMSD values of the heavy atoms of BXA stabilizes after 40 ns, with the average of 2.95 ± 0.37 Å for frames between 40 and 100 ns (ESI Fig. S2d†).

MM-GBSA has been used to estimate the ligand binding energy with the target protein structure and the method has been found to be useful in improving the predicted results from molecular docking based virtual screening studies.^84^ Thus, we calculated the binding energies of the top 5 phytochemical inhibitors identified in this study with the endoN using MM-GBSA method (Table 1; Methods). It is to be noted that the binding energies calculated using MM-GBSA method represent relative binding energies and do not include full entropy contributions.^85–87^ The inhibitor 2′-deoxymugineic acid (L2) has the lowest MM-GBSA based binding energy value of −44.83 ± 3.33 kcal mol^−1^, followed by epoxyoleic acid (L5) with binding energy value of −40.66 ± 7.67 kcal mol^−1^, traumatic acid (L3) with binding energy value of −38.00 ± 5.14 kcal mol^−1^, gamma-glutamylaspartic acid (L1) with binding energy value of −27.23 ± 5.57 kcal mol^−1^, and betalamic acid (L4) with binding energy value of −16.61 ± 3.96 kcal mol^−1^. Although the known inhibitor BXA was found to have a docking binding energy value of −7.3 kcal mol^−1^ with the endoN, its MM-GBSA based binding energy was found to be −41.35 ± 2.44 kcal mol^−1^.

## Conclusions

Several pandemics and epidemics which led to huge loss of human life worldwide have been recorded in the history. Presently, the ongoing COVID-19 pandemic has led to more than 5 million deaths and has affected several hundred million globally. This re-emphasizes the need for increased scientific research towards studying emerging pathogenic viruses with pandemic potential such as SFTSV and to develop drugs against them. After the first documented cases of SFTSV infection in 2009, several outbreaks of SFTS have been subsequently reported in multiple East Asian countries including China, South Korea and Japan.^3^ With fatality rates ranging from 2.8% to 47%^88^ and the gradual worldwide spread of the SFTSV vector,^3^ SFTS poses a serious threat to global public health. Thus, urgent attention is required towards the discovery of novel vaccines and drugs against SFTSV. However, there are presently no approved antiviral drugs or vaccines against SFTSV.

A number of natural product or natural product derived molecules have been successfully developed as drugs and have contributed immensely to the approved drug space. Specifically, medicinal plants are a rich source of diverse bioactive molecules that can be potentially harnessed for the development of antiviral drugs against emerging pathogenic viruses such as SFTSV. The endoN is a key component involved in initiation of viral RNA transcription in SFTSV, and thus, is an ideal drug target. In this study, we have implemented a four-stage virtual screening workflow starting with a small molecule library of 14 011 phytochemicals from Indian medicinal plants. Briefly, 10 510 drug-like phytochemicals were filtered from 14 011 phytochemicals (stage 1), which were used for performing docking against 26 structures of the endoN to consider the conformational heterogeneity of the active site. Filtration post docking based on binding energy led to a subset of 178 phytochemicals (stage 2). The above set of phytochemicals were further filtered based on non-covalent interaction between the ligand and the key binding site residues, resulting in the identification of 26 phytochemicals (stage 3). Finally, using chemical similarity, chemical class and toxicity prediction, 5 potential phytochemical inhibitors of the endoN namely, gamma-glutamylaspartic acid (L1), 2′-deoxymugineic acid (L2), traumatic acid (L3), betalamic acid (L4) and epoxyoleic acid (L5) were identified (stage 4). In the virtual screening workflow, we integrated several computational approaches such as ensemble docking, MD simulations, drug-likeness filter, protein–ligand non-covalent interaction filter, toxicity filter and chemical similarity network to identify the 5 potential phytochemical inhibitors (L1–L5) of the endoN. Further, we have provided the plant source, chemical structure, chemical classification, physicochemical properties, drug-likeness properties, ADMET properties and toxicity prediction of the potential phytochemical inhibitors of the enodN, to aid future experimental analysis towards development of drugs against SFTSV. We would however like to emphasize that further *in vitro* and/or *in vivo* experiments are required to validate the anti-SFTSV activity of the potential phytochemical endoN inhibitors identified in this study. Previously, an *in vitro* screening of the US FDA approved drugs identified five compounds with anti-SFTSV activity, amongst which hexachlorophene was found to be a viral entry inhibitor with predicted binding to Gc glycoprotein.^89^ To the best of our knowledge, virtual screening of a natural product library against the endonuclease domain of SFTSV L polymerase has not been attempted prior to this study. Thus, in conclusion the potential phytochemical inhibitors of SFTSV endoN identified in this study can be taken up for development of anti-SFTSV drugs, and the virtual screening protocol implemented here can serve as a template for screening natural product libraries against drug targets with consideration to the conformational heterogeneity of the active site.

## Conflicts of interest

The authors declare that they have no conflicts of interest.

## Supplementary Material

RA-012-D1RA06702H-s001

RA-012-D1RA06702H-s002
