# Supplementary material for: Virtual screening of phytochemicals from Indian medicinal plants against the endonuclease domain of SFTS virus L polymerase
Source: RSC Adv. 2022 Feb 22;12(10):6234–47. doi: 10.1039/d1ra06702h (PMC8982020; doi:10.1039/d1ra06702h)
Supplement: RA-012-D1RA06702H-s001 [file RA-012-D1RA06702H-s001.pdf]

Electronic Supplementary Material (ESI) for RSC Advances.
This journal is © The Royal Society of Chemistry 2022

Supplementary Information

Figures S1-S2

for

Virtual screening of phytochemicals from Indian medicinal plants against endonuclease domain of SFTS Virus L polymerase

R. P. Vivek-Ananth [a,b], Ajaya Kumar Sahoo [a,b], Ashutosh Srivastava [c,*], Areejit Samal [a,b,*]

[a] *The Institute of Mathematical Sciences (IMSc), Chennai 600113, India*

[b] *Homi Bhabha National Institute (HBNI), Mumbai 400094, India*

[c] *Discipline of Biological Engineering, Indian Institute of Technology Gandhinagar, Gandhinagar 382355, India*

* Authors to whom correspondence should be addressed:

Areejit Samal (asamal@imsc.res.in) or

Ashutosh Srivastava (ashutosh.s@iitgn.ac.in)

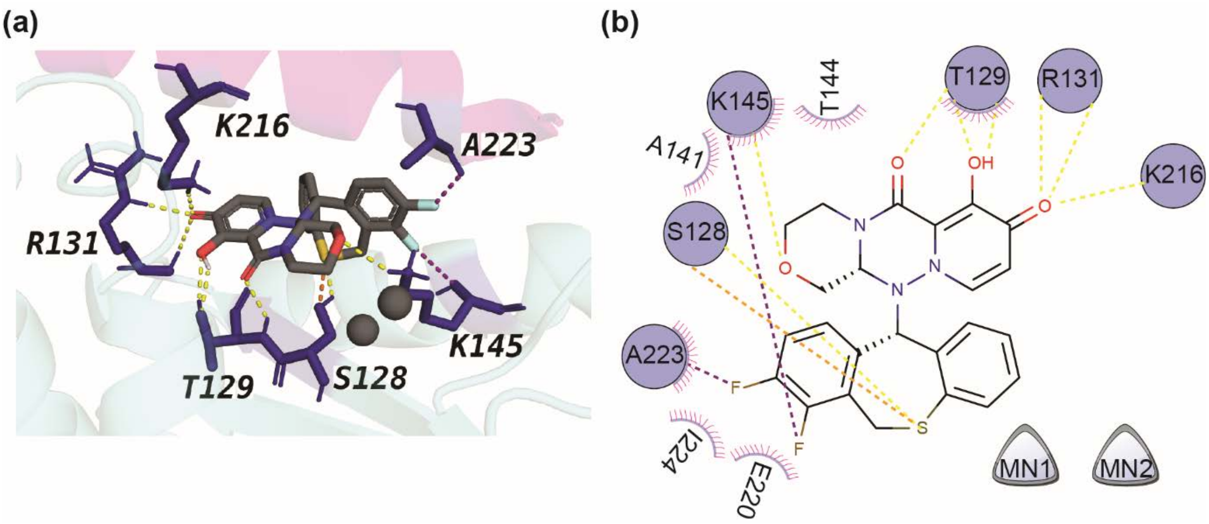

Figure S1: (a) Cartoon and (b) 2D representation of the interactions between the known endonuclease inhibitor Baloxavir acid (BXA) and the protein residues in the best docked pose with the endoN. In (a), the carbon, oxygen, nitrogen, fluorine and sulfur atoms are in dimgrey, red, blue, cyan and dark yellow, respectively. The protein residues involved in non-covalent interactions with BXA are shown as sticks colored in deepblue. The Mn^{2+} ions are shown as grey colored spheres. In (b), the protein residues involved in non-covalent interactions with BXA are shown as circles colored in deepblue. The protein residues involved in hydrophobic interactions with BXA are shown as short circle segments with spike. The Mn^{2+} ions are shown as triangles. In both (a) and (b), the hydrogen bond interactions are shown as yellow colored dashed lines, the halogen bond interactions are shown as purple colored dashed lines and the chalcogen interactions are shown as orange colored dashed lines. In the schematic figure (b), the protein residues involved in non-covalent interactions with BXA have been manually placed around the 2D chemical structure of BXA.

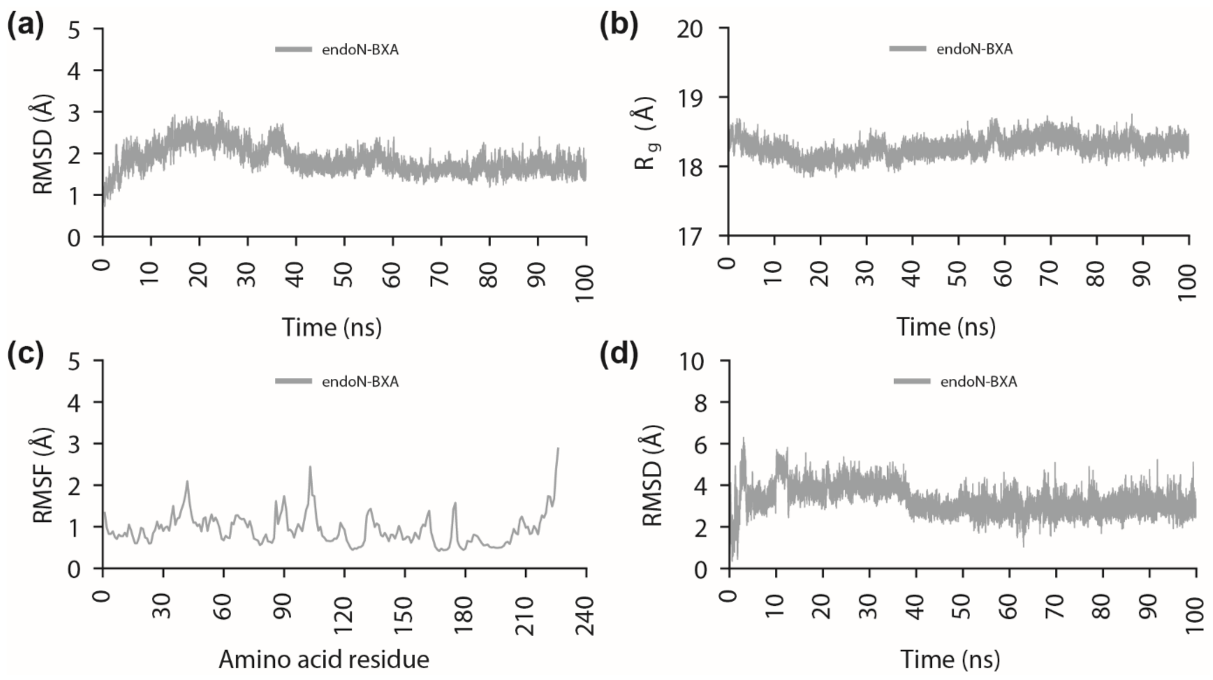

Figure S2: Analysis of the trajectories from the 100 ns MD simulations of the protein-ligand docked complex of Baloxavir acid (BXA) with the endoN. (a) RMSD of the Cα atoms of all protein residues and Mn^{2+} ions. (b) Radius of gyration (R_g) of the complete protein structure. (c) RMSF of the Cα atoms of all protein residues. (d) RMSD of the heavy atoms of the ligand BXA.
